# Massive Lipomatosis of the Small Intestine Causing Intussusception

**DOI:** 10.1155/2019/9701478

**Published:** 2019-12-20

**Authors:** Michael Pagacz, Irvin Willis, John Alexis

**Affiliations:** ^1^A.M. Rywlin, MD Department of Pathology, Mount Sinai Medical Center, Miami Beach, FL, USA; ^2^Department of General Surgery, Mount Sinai Medical Center, Miami Beach, FL, USA; ^3^Herbert Wertheim College of Medicine, Florida International University, Miami, FL, USA

## Abstract

Lipomatosis is a rare condition characterized by diffuse, unencapsulted adipose tissue deposition. Intestinal involvement is rare, and presentation as intussusception is rarer still. We report a 40-year-old man who presented with abdominal pain and fecal urgency. Abdominal CT scan showed a protuberant ileo-cecal valve, with intussusception of the ileum into the cecum. The mucosal surface of the resected bowel was bulbous and protuberant, showing loss of mucosal folds, and there was an 8 × 5 × 5 cm mass prolapsing into the ileo-cecal valve. Microscopically there was abundant adipose tissue in the submucosa with an unremarkable mucosa. The patient recovered uneventfully with only occasional cramping in the left abdomen.

## 1. Introduction

Intestinal lipomatosis is a disease of unknown etiology which may remain asymptomatic or present with complications such as intussusception. It is exceedingly rare, and the presentation as intussusception is rarer still [1]. We report a case of a symptomatic man with intestinal lipomatosis of the small bowel presenting as intussusception.

## 2. Case Report

**Figure 1:**
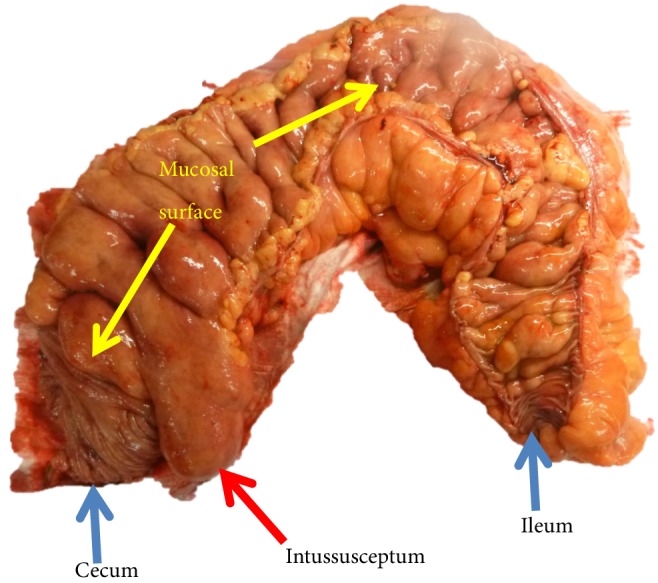
Opened bowel with mucosal surface.

**Figure 2:**
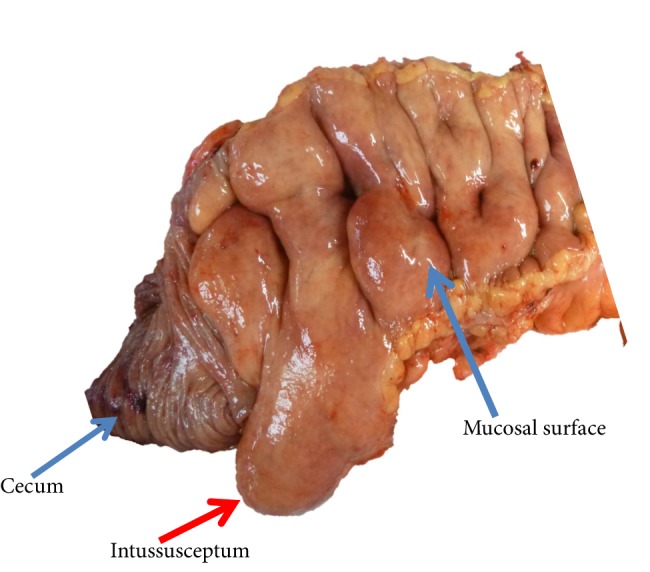
Intussusceptum at ileocecal valve.

**Figure 3:**
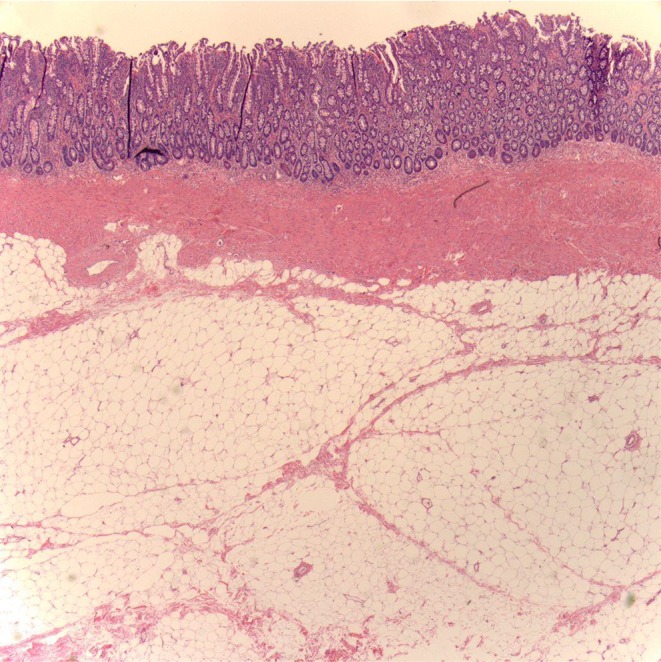
Intussuscepted lipomatous mass H&E 4x.

The patient is a 40-year-old man with a history of gastroesophageal reflux disease and a family history of colon cancer. He presented with severe abdominal pain, fecal urgency, and episodes of diarrhea. He had lost 22 lbs over two months. An abdominal CT and colonoscopy showed a severely lipomatous, bulbous, and protuberant ileo-cecal valve, with intussusception of the ileum into the cecum. Exploratory laparotomy revealed marked enlargement of the ileum due to massive fat deposition, and adhesions between the ascending colon and the right lateral lower abdominal wall. A dilated segment of the ileum and cecum was surgically resected, which demonstrated a bulbous, protuberant, abnormally smooth mucosal surface in the ileum, sharply demarcated from normal large intestinal mucosa at the ileo-cecal valve (Figure 1). There was an 8 × 5 × 5 cm mass protruding through the ileo-cecal valve into the cecum (Figure 2). At the proximal end of the resected specimen there was a short (4 cm) transition from normal small intestinal mucosa to the abnormal, smooth, bulbous mucosa. Microscopically, there was extensive infiltration of mature adipose throughout the submucosa (Figure 3).

## 3. Discussion

This is an unusual case of lipomatosis of the small intestine presenting as an intussusception of a submucosal mass into the ileo-cecal valve. 90% of cases are localized in the intestinal submucosa, occasionally with reaching muscularis propria, and 10% are subserosal [1]. The etiology of lipomatosis is unknown. Theories include embryonic displacement of adipose tissue, postchemotherapeutic fat deposition, and chronic irritation such as chronic inflammatory bowel disease, low-grade infection, and hamartomatous syndromes [2].

Only fifteen documented cases of diffuse intestinal lipomatosis exist and only two documented cases of intussusception caused by diffuse lipomatosis [1]. Most patients in reported cases of intestinal lipomatosis were asymptomatic; however, some presented with subacute intermittent obstruction, colonic perforation, and intussusception, the rarest of complications [3]. Early diagnosis of adult intussusception is difficult because most cases present with nonspecific signs and symptoms and may present in an acute, subacute or chronic manner. The classic triad of intermittent abdominal pain, currant jelly stools, and a palpable tender mass seen in children is rarely present in adults. However, in adults, nausea, vomiting, gastrointestinal bleeding, changes in bowel habits, and abdominal distension are more common [4].

The most useful imaging techniques for evaluating fatty lesions of the intestine are CT scan and ultrasound. Lipomas of the small intestine may appear on CT scan as round, homogeneous, well-circumscribed masses with fat density, while on ultrasonography they usually appear as highly echoic masses. Ultrasonography of intussusception typically shows a multilayered appearance consisting of the alternating hyperechoic and hypoechoic concentric rings that represent alternating layers of mucosa, bowel wall, and mesenteric fat in cross section. The sensitivity and specificity of ultrasonography and CT are similar. Ultrasonography is more useful for the initial diagnosis of intussusception as it is more readily available, enabling it to be used in emergent situations [5].

The prognosis of intussusception is quite variable, depending on the cause. Mortality for adult intussusception is 8.7% in cases with benign lesions, and 52.5% in cases with malignant neoplasms [6]. Invasive management of lipomatosis is not advised unless complications arise such as intussusceptions, obstruction, bleeding, or perforation leading to peritonitis in which case prompt surgical intervention is essential to prevent serious complications such a bowel ischemia and perforation [1, 7].

## 4. Conclusion

Intussusception in adults is an uncommon condition invariably associated with an underlying cause which needs pathological examination to arrive at a definitive diagnosis. Small bowel lipomatosis is a rare condition that can cause intussusception presenting as obstruction. Prompt diagnosis is crucial as delay in treatment may have serious consequences.
